# Differential expression of folate receptor 1 in medulloblastoma and the correlation with clinicopathological characters and target therapeutic potential

**DOI:** 10.18632/oncotarget.15480

**Published:** 2017-02-18

**Authors:** Hailong Liu, Qianwen Sun, Mingshan Zhang, Zhihua Zhang, Xinyi Fan, Hongyu Yuan, Cheng Li, Yuduo Guo, Weihai Ning, Youliang Sun, Yongmei Song, Chunjiang Yu

**Affiliations:** ^1^ Department of Neurosurgery, Sanbo Brain Hospital Capital Medical University, Beijing, P.R. China; ^2^ State Key Laboratory of Molecular Oncology, National Cancer Center/Cancer Hospital, Chinese Academy of Medical Sciences and Peking Union Medical College, Beijing, P.R. China; ^3^ Department of Neurology, Qilu Hospital Shandong University, Jinan, P.R. China; ^4^ School of Basic Medical Science, Capital Medical University, Beijing, P.R. China

**Keywords:** medulloblastoma, folate receptor 1, prognosis, biomarker, therapeutic target

## Abstract

Medulloblastoma is the most common malignant brain tumor in children. Folate receptor 1 (Folr1) was abundantly expressed in some epithelial malignancies. However the expression profile and the role of clinicopathological significance and therapeutic target potential in medulloblastoma still remain elusive. Currently we detected the expression of Folr1 in medulloblastoma and identified the diagnostic application by evaluating the clinical, pathological and neuroimaging values. Then we developed a target therapeutic compound with Folr1, which exhibited promising efficiency in treatment of medulloblastoma. Folr1 expression was up-regulated in medulloblastoma and positively correlated with percentage of Ki-67 and MMP9 labeling, pathological subtypes, serum Folr1 levels and CSF spreading on MRI. The level of serum Folr1 showed rational sensitivity and specificity in predicting histological subgroups. Strong Folr1 expression was recommended as the independent value regarding the prognosis of patients with medulloblastoma. Folr1 targeted therapy attenuated the tumor growth and metastasis with down-regulation of MMPs proteins and activation of apoptosis. Immunostaining analysis in the xenograft samples showed the decreased Ki-67 and MMP9 index providing the strong evidences that Folr1 targeted application can suppress the proliferation and invasion. Our findings uncovered in Folr1 a predictive candidate and therapeutic target for medulloblastoma.

## INTRODUCTION

Medulloblastoma (MB) is the most common malignant brain tumor in children, accounting for about 20% of children with intracranial tumors [1, 2] and about 50% of patients with metastasis along the leptomening via circulation of CSF [3]. According to the WHO classification [4, 5] and International Medulloblastoma Working Group [6], MB mainly contains four histological subgroups, including desmoplastic/nodular MB (D/N MB), classic MB, large cell MB and anaplastic MB. Clinically, the presence of large cell and anaplastic MB, or Group 3/4 MB is added into the high-risk criteria whereas desmoplastic/nodular or WNT characteristic is a hopeful prognostic index [5, 7]. Children who are more than 3 years old with the focal tumor at first diagnosis and the less than 1.5 cm^3^ residual tumor after operation will be classified in the average-risk group [8, 9]. However, these features are only involved in the postsurgical prognosis and so far no items in blood or tissue samples have been regarded as the non-invasive biomarkers at the early stage.

Folate receptor 1 (Folr1) is a glycosylphosphati- dylinositol protein anchored to cell membranes which can transport folate or its derivants into cells via the endocytic process [10, 11]. Folr1 over-expresses in a wide range of epithelial malignant cancers, such as ovarian, lung and breast carcinomas [12–14], also in some neruoepithelial tumors [15–18]. Our previous research showed that Folr1 expressed extensively in glioblastoma (GBM) and the level was positively correlated with malignancy grade [18]. Folr1 also locates in some normal tissues including placenta, kidney tubules and choroid plexus, strictly on the apical surface of a limited subset of polarized epithelial cells [11, 19, 20]. Based on the unique high expression of Folr1 in malignancies and restricted expression in normal tissues, cytarabine (Ara-C) binding to folic acid (FA) has been developed as a targeted therapeutic compound which can be delivered into tumor cells through its recognition and combination with Folr1 specifically in our previous study [21]. Folate receptor 1-targeted cytarabine (Folr1-Ara-C) displayed promising advantages in two ways: (1) Improve the targeting therapeutic efficiency and bypass drug resistance by binding to the high affinity Folr1. (2) Reduce the dosage of Folr1-Ara-C versus Ara-C which doesn't cause obvious side effects but inhibits tumor growth. However, the biological role of Folr1 and its clinical significance in targeted therapeutic strategy for MB still remain unclear. The recent research throws light on the diagnostic and prognostic value of Folr1, as well as the therapeutic implications of Folr1-Ara-C on MB.

In this study, we showed that Folr1 levels increased progressively in different histological subtypes of MB, and that Folr1 correlated with clinical, pathological, neuroimaging features and prognosis in MB patients. Because the serum Folr1 protein secreted by tumor cells in soluble form has been referred as a biomarker of prognosis in ovarian cancer patients [22, 23], we evaluated the soluble Folr1 in serum or CSF as an indicator for MB. Then we continuously detected the effects of Folr1-Ara-C on the proliferation, mobility and apoptosis of MB cells *in vitro* and *in vivo*, and studied the mechanisms of this compound. These data revealed a critical role for Folr1 in early diagnosis of MB and pointed to Folr1 as a potential therapeutic target for this devastating disorder. To the best of our knowledge, it is the first novel study regarding the diagnostic and target therapeutic implications of Folr1 in MB.

## RESULTS

### Expression of Folr1 in MB

**Figure 1:**
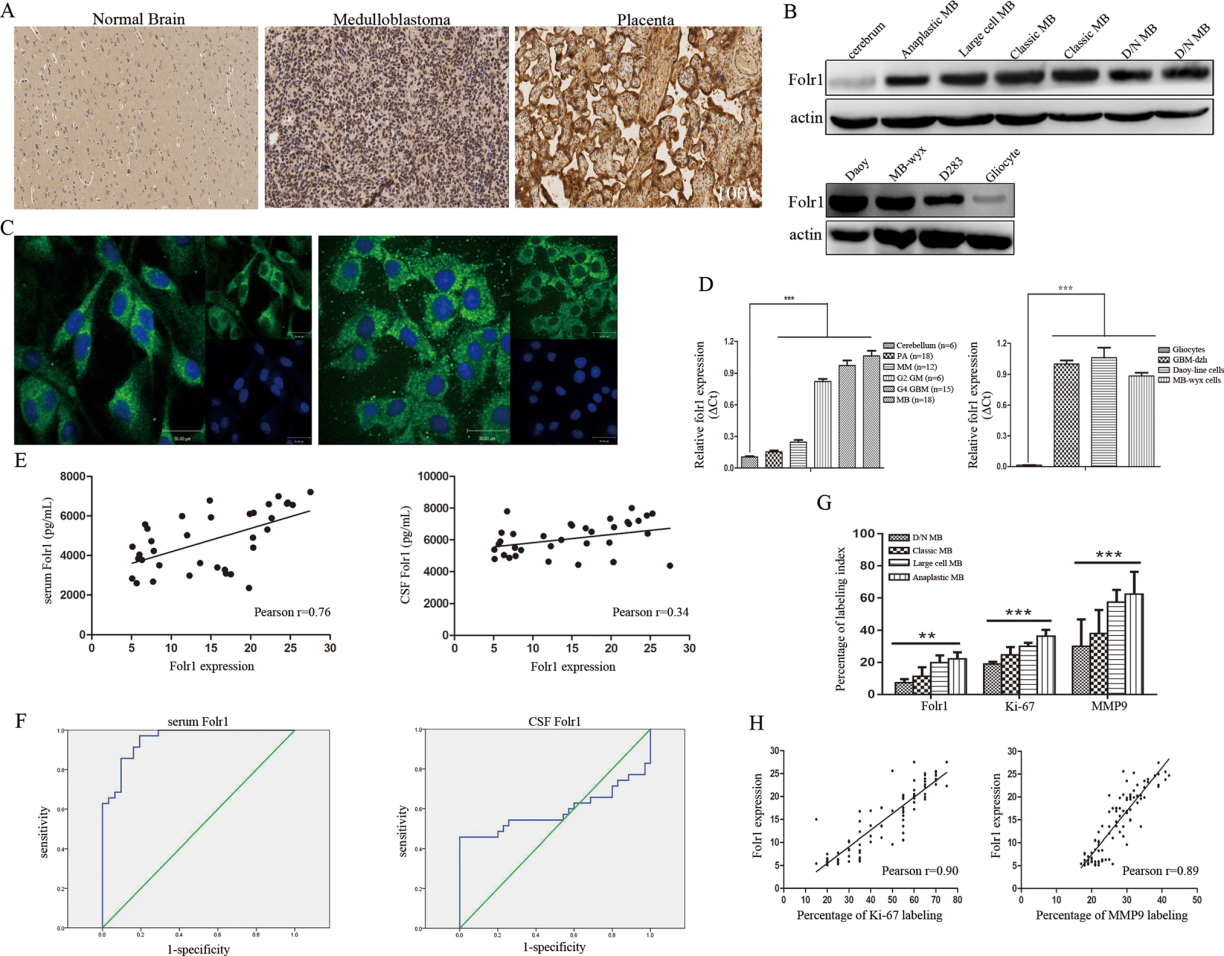
Folr1 expression in MB tissues and cells (**A**) Representative IHC images of Folr1 expression in cerebrum, human MB tumor specimens and placentas in low-magnifying fields (100 ×). (**B**) Immunoblotting analysis of the Folr1 levels in human MB specimens (up) and tumor cells (down). (**C**) Representative confocal images of Folr1 expression in Daoy cells (left) and MB-wyx cells (right). (**D**) The relative mRNA levels of *folr1* in different tissues (left) and cells (right). Bars are *mean ± SD* from at least three independent experiments. ***P < 0.001. (**E**) Statistical graph of correlation between Folr1 expression and serum (left) or CSF (right) Folr1 by Pearson analysis. (**F**) ROC curve of serum (left) and CSF (right) Folr1 for evaluating the biomarker significance for MB patients. (**G**) Statistical graph of Folr1, Ki-67 and MMP9 staining detected by IHC in four subtypes of MB. (**H**) Positive correlation between Folr1 expression and Ki-67 (left) or MMP9 (right) labeling was shown by Pearson analysis.

In a total of 95 MB subjects: 4 (4.21%) were D/N MB, 60 (63.16%) were classic MB, 12 (12.63%) were large cell MB and 19 (20.00%) were anaplastic MB. The expression of Folr1 was detected by IHC. As shown in Supplementary Table 1, Folr1 was weak positive in 33 MB (34.74%), moderate positive in 17 MB (17.89%), strong positive in 19 MB (20.00%) and very strong positive in 26 MB (26.37%). In normal brain tissues, 6 (100.00%) cases showed negative expression and 3 (100.00%) cases of placentas displayed very strong expression (Figure 1A and Supplementary Figure 1A). Folr1 exhibited significant amounts of staining in human MB specimens and tumor cells (Figure 1B and 1C). The relative mRNA of *folr1* showed markedly increased levels in MB (about 10.0-fold compared with normal tissues), but little expression in benign tumors like pituitary adenomas and meningiomas (about 1.5-fold and 2.0-fold respectively, Figure 1D).

### Results of serum and CSF Folr1 in MB patients

Data of serum and CSF Folr1 in 35 patients with MB and 35 patients with meningiomas (WHO-I) were shown in Supplementary Figure 1B. High levels of serum Folr1 were observed in large cell and anaplastic MB but not in patients with benign control and D/N MB. Meningiomas through anaplastic MB, results of serum Folr1 appeared gradually increasing and the difference was statistically significant. Whereas similar trend was not obtained in CSF group (Supplementary Figure 1B).

Then the correlation between Folr1 expression in human MB specimens and serum/CSF Folr1 was investigated by Pearson analysis (Figure 1E). There existed a positive tendency between serum Folr1 and Folr1 profile in specimens. However, the Pearson index in CSF Folr1 was lower than 0.50, suggesting that only a low correlation existed between the two features.

Receiver operator characteristic (ROC) analysis could provide tools to select the possible optional models in the natural way to highlight diagnostic decision making. Here ROC curve was utilized to evaluate the importance of serum or CSF Folr1 in indicating the MB diagnosis like the function of AFP in hepatocellar carcinoma (Figure 1F). Summary analysis of ROC curve was shown in Supplementary Table 2. Area under the curve (AUC) of serum and CSF Folr1 was 0.95 versus 0.58, which figured out that serum Folr1 contributed more to MB diagnosis, but not the CSF Folr1. Youden's index was suggested as the way of capturing the performance of a diagnostic test. Its values ranged from -1 to 1, indicating that the tests had more significance with the values closer to 1. Youden's index with values of 0.78 and 0.46 in current research indicated that serum Folr1 showed better performance. Serum Folr1 exhibited absolutely higher values of sensitivity, specificity and accuracy and presented higher levels of NPV and PLR compared with the CSF group, as well as lower counts of PPV and NLR. The *Kappa* value in serum Folr1 group was 0.77 compared with the value of 0.46 in CSF group (Supplementary Table 2). Taken together, these results revealed that serum Folr1 could be recommended as a potential non-invasive biomarker to predict MB status.

### Correlation of Folr1 expression with clinicopathological and neuroimaging features

The correlation between Folr1 expression and clinical features (gender, age and prognosis), pathological features (Ki-67, MMP9 labeling and subtypes) as well as neuroimaging features (enhancement, peritumor edema, maximum diameter and CSF spreading on MRI) was further analyzed. Ki-67 labeling was identified as the low percentage (< 29.0%) in 22 MB (23.16%), the moderate percentage (30.0–59.0%) in 42 MB (44.21%) and the high percentage (> 60.0%) in 31 MB (32.63%). MMP9 expression was weak positive in 12 MB (12.63%), moderate positive in 46 MB (48.42%) and strong positive in 37 MB (38.95%) (Supplementary Figure 2 and Supplementary Table 1). The three markers showed an extensively higher expression in large cell and anaplastic MB than those in the other two subtypes (Figure 1G). Then by Logistic regression analysis, odd ratio (OR) values of the threes were 1.03, 1.15 and 1.10 respectively supporting that they performed as the risk factors (Supplementary Table 1). In addition, the relationship between Folr1 expression and Ki-67 or MMP9 labeling was analyzed by Pearson analysis showing the positive tendency (Figure 1H).

Among the 95 patients, CSF spreading on MRI occurred in 55 cases (57.89%) and Folr1 expression was positively associated with this neuroimaging character (Supplementary Table 1 and Supplementary Figure 3). The OR value presented more than 2.00 suggesting that it was also a risk factor (Supplementary Table 1). However, no correlation was found in other clinical and radiological variables including factors of age, prognosis and enhancement, peritumor edema and maximum diameter on MRI as listed in Supplementary Table 1. We next assessed the potential prognostic impacts of these properties by univariate and multivariate Cox survival analysis showing that age, CSF spreading on MRI, high percentage of Ki-67, strong expression of MMP9 and Folr1 as well as anaplastic subtype could be regarded as the independent prognostic values (Supplementary Table 3). Thus, MB possessing high expression of Folr1 was correlated with the proliferative and invasive indexes, such as Ki-67, MMP9 labeling and CSF spreading on MRI.

### Association of Folr1 expression with prognosis of MB patients

**Figure 2:**
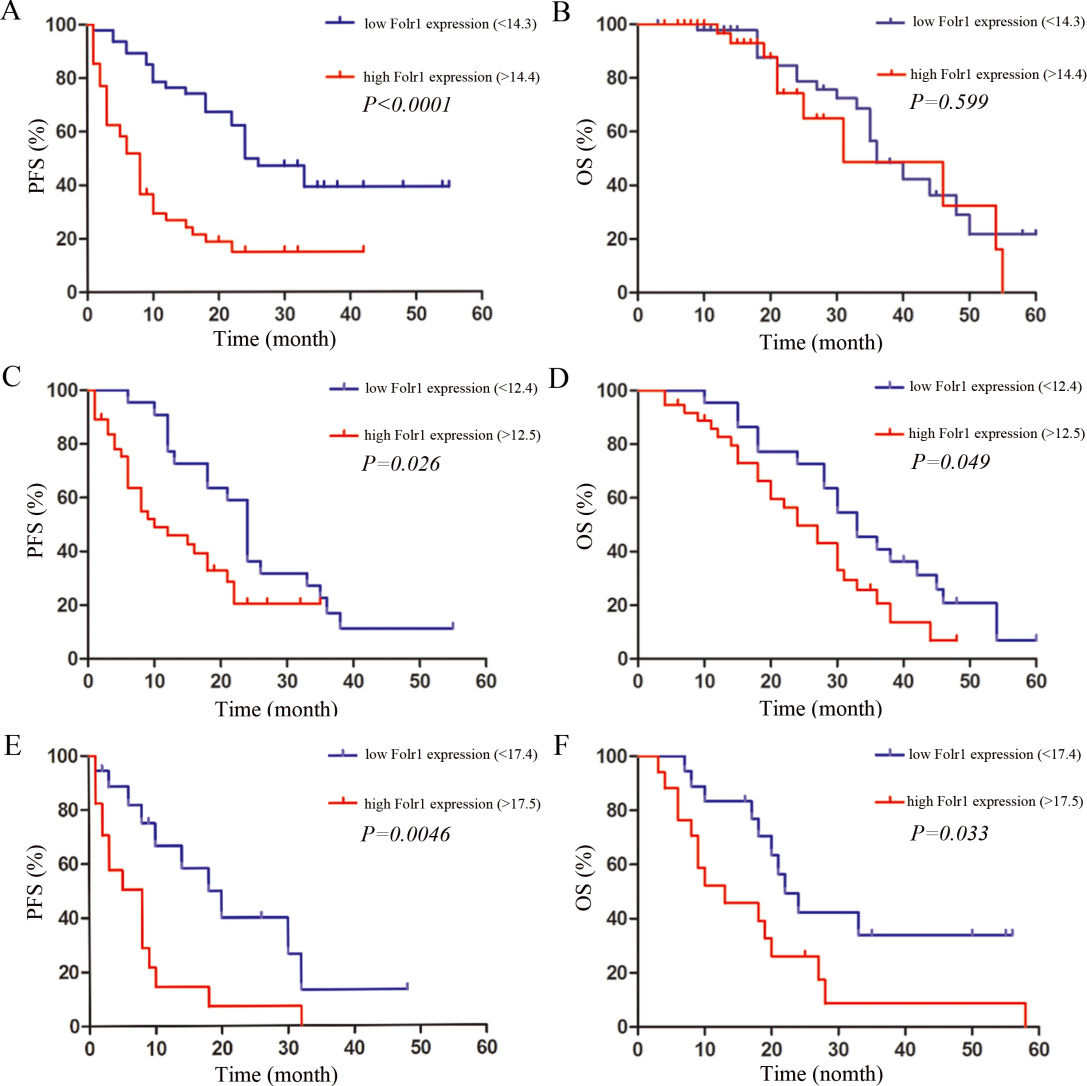
The association between prognosis and Folr1 expression in MB patients (**A**–**B**) The PFS (A) and OS (B) in all the MB patients with the average Folr1 expression defined as the value of 14.3. (**C**–**D**) The PFS (C) and OS (D) in the average-risk group with the average Folr1 expression defined as the value of 12.4. (**E**–**F**) The PFS (E) and OS (F) in the high-risk group with the average Folr1 expression defined as the value of 17.4. Patients with Folr1 levels higher or lower than average expression are considered as high or low, respectively.

In the cohort of 95 cases, the median follow-up time was 32.6 months (range, 3.0–66.0 months), 32 (33.68%) patients have been alive and there were 63 (66.32%) death due to MB during the follow-up periods. Then the association between Folr1 expression and progression-free survival (PFS) or overall survival (OS) was assessed by Kaplan-Meier analysis in terms of survival. Patients with lower Folr1 expression (Folr1 < 14.3, this score was the average of all patients) demonstrated a dramatically longer PFS than those with higher levels (Figure 2A). However, no difference existed in OS between the lower and higher group (Figure 2B). As shown in Supplementary Table 4, factors of age, CSF spreading, percentage of Ki-67 and MMP9 labeling and pathological subtypes also affected the OS.

Furthermore, all the 95 cases were separated into the average-risk and high-risk group according to the current clinical risk grades for MB [9, 24]. In the average-risk group, the patients with lower expression (Folr1 < 12.4, this score was the average in average-risk group) showed more promising PFS (Figure 2C) and OS (Figure 2D) than those with higher Folr1. Similarly, the patients in the high-risk group with lower Folr1 expression also presented the better PFS (Figure 2E) and OS (Figure 2F). Therefore, these data suggested that expression of Folr1 protein made sense to predict the postsurgical survival of MB patients.

### Effects of Folr1-Ara-C on cell proliferation

**Figure 3:**
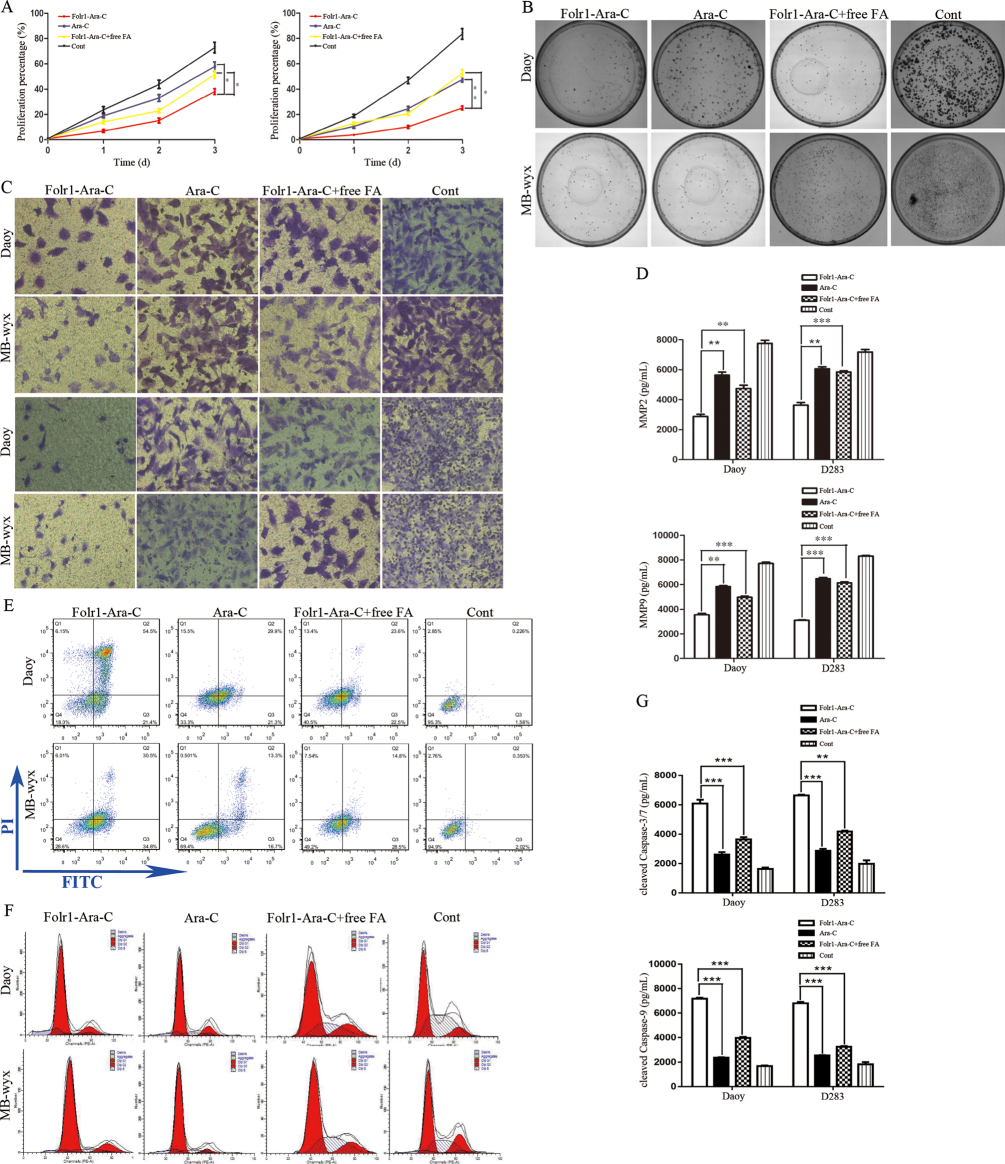
Effects of Folr1-Ara-C on on the proliferation, mobility and apoptosis of MB cells (**A**) Daoy (left) and MB-wyx (right) cells were treated with the indicated concentration of Folr-Ara-C, Ara-C, Folr1-Ara-C+free FA and control for 8 h, respectively. Then cell proliferation was measured by MTS assay. (**B**) Colony formation assay of Daoy (up) and MB-wyx (down) cells was performed in the presence of Folr-Ara-C, Ara-C, Folr1-Ara-C+free FA and control for 8 h and cells were stained with 0.1% crystal violet. (**C**) Cell migration (up) and invasion (down) were evaluated by the transwell chamber assay without or within the membrane coated with Matrigel. (**D**) Secretion of MMP2 (up) and MMP9 (down) after 8 h of incubation with the indicated agents was analyzed by ELISA. (**E**) Representative images of cell apoptosis by FCM assay were shown. (**F**) Impacts of Folr1-Ara-C on Daoy (up) and D283 (down) cell cycle was analyzed by measuring the PI binding activity. (**G**) Cell apoptosis was evaluated by the cleaved Caspase-3/7 (up) and -9 (down) activity, respectively. Bars are *mean ± SD* from at least three independent experiments. *P < 0.05, **P < 0.01, ***P < 0.001.

The folate derivative, Folr1-targeted Ara-C could be transported into tumor cells by recognizing and combining with the unique Folr1 profile specifically. To explore the anti-proliferative effect of Folr1-Ara-C on MB cells, MTS assay was performed with the IC_50_ value of Folr1-Ara-C reported in our previous research [21]. Incubation with 2.00 × 10^−3^ mmol/L Folr1-Ara-C significantly reduced cell growth compared with the Ara-C group. When Folr1 was blocked by free FA, the inhibitory capability of Folr1-Ara-C was markedly decreased (Figure 3A). Anchorage-dependent colony formation assay was performed to evaluate the repressing efficiency of Folr1-Ara-C on proliferation, showing that Folr1-Ara-C could produce greater cytotoxicity than Ara-C in Daoy cells. The similar results were also observed in MB-wyx cells (Figure 3B and Supplementary Figure 4A).

### Impacts of Folr1-Ara-C on cell mobility and apoptosis

**Figure 4:**
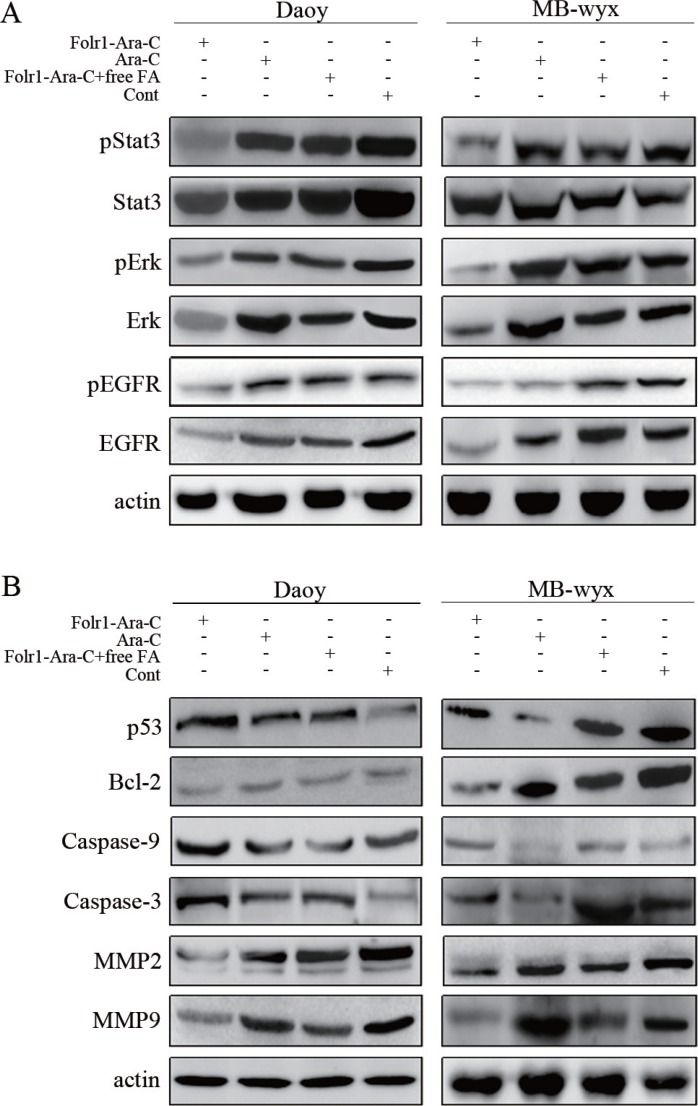
Molecular mechanisms of Folr1-Ara-C functions (**A**) Targeted effects of Folr1-Ara-C on the inhibition of EGFR, pEGFR, Erk, pErk and pStat3 in Daoy and MB-wyx cells after 8 h incubation. Lysates were analyzed for EGFR/pEGFR, pErk/Erk and pStat3/Stat3 antibodies using immunoblotting. β-actin was used as the protein loading control. (**B**) Immunoblotting analysis of MMP2, MMP9, Caspase-3/-9, Bcl-2 and p53 in Daoy and MB-wyx cells incubated with indicated agents, respectively. β-actin was used as the protein loading control.

To investigate the impacts of Folr1-Ara-C on migration and invasion, tumor cells were generated with 2.00 × 10^−3^ mmol/L these agents for 8 h and then transwell assay was assessed. As demonstrated in Figure 3C and Supplementary Figure 4B, Folr1-Ara-C dramatically attenuated the ability of Daoy cells to migrate and invade. According to the above IHC data, we further measured the concentration of MMP2 and MMP9 in the supernatant of MB cells after administration with the indicated agents by ELISA. Folr1-Ara-C incubation for 8 h inhibited more effectively MMP2 secretion than other groups with the treatment of Ara-C, Folr1-Ara-C+free FA and PBS control (Figure 3D up). Simultaneously, secretion of MMP9 was also suppressed by Folr1-Ara-C to a greater degree (Figure 4D down). Within the free FA (1.0 mmol/L) competing For1-Ara-C for Folr1 binding, the inhibitory capability of For1-Ara-C was decreased obviously. The similar results were obtained in MB-wyx cells.

Then we sequentially detected the apoptosis in Daoy and D283 cells treated with the agents by conducting FCM and cleaved caspase -3/7 and -9 activity assays. As shown in Figure 3E and Supplementary Figure 4C, Folr1-Ara-C could induce Daoy cell apoptosis up to 73.15%, whereas Ara-C only induced to 51.22% (Figure 3E). The results of the cleaved caspase-3/7 and -9 activity assay showed that a similar increase of apoptotic rate was reflected in For1-Ara-C group (Figure 3G). Ara-C was known to be one kind of cell-cycle specific drugs by blocking S phase [25]. We further investigated the functions of Folr1-Ara-C on cell cycle by measuring the PI binding activity. Figure 3F showed that Folr1-Ara-C led more Daoy cells to be blocked in S phase and Folr1-Ara-C with free FA analogously down-regulated the blocking ability substantially about 17%. Similar results were shown in D283 cells (Supplementary Figure 4D). Thus, Folr1-Ara-C contributed more to anti-tumor activity regarding the proliferation, mobility and apoptosis.

### Molecular mechanisms of Folr1-Ara-C functions

To explore the Folr1-Ara-C mediated oncogenic pathways, such as Erk, Stat3 and PI3K/Akt pathways, immunoblotting assay was performed to analyze the activity of the major molecules within these signaling pathways, including total Erk, p-Erk, total Akt, p-Akt, total Stat3, p-Stat3 total EGFR and p-EGFR after treatment of the indicated agents as above described. As shown in Figure 4A, decreased levels of total Erk, total EGFR, p-EGFR, p-Erk and p-Stat3, but not Akt (data not shown) or total Stat3 were observed in Daoy cells after Folr1-Ara-C management compared with the Ara-C group. Similar results were obtained in MB-wyx cells (Figure 4A).

The mechanistic investigation was continuously further conducted underlying the regulation of invasion and apoptosis in MB. Folr1-Ara-C could distinctly diminish the levels of invasion-associated proteins, such as MMP2 and MMP9, as well as upregulate the degrees of Bcl-2 and p53 protein (only in Daoy cells, Figure 4B).

### Therapeutic application of Folr1-Ara-C in mouse xenografts

**Figure 5:**
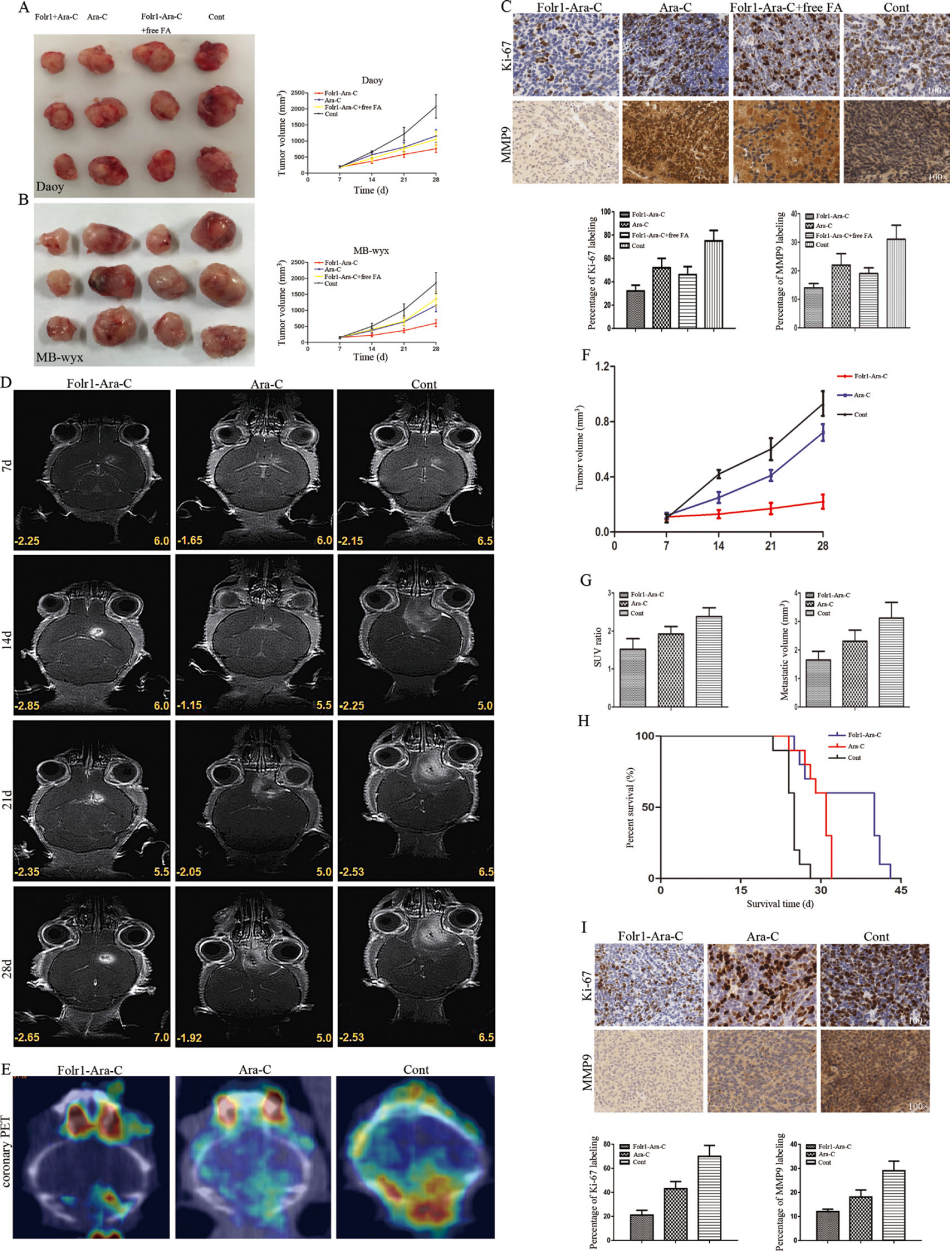
Application of Folr1-Ara-C on subcutaneous and intracranial xenografts derived from MB cells (**A**–**B**) Daoy (up) and MB-wyx (down) cells were subcutaneously transplanted into nude mice respectively and representative images were shown as the targeted therapeutic effects of Folr1-Ara-C. (**C**) Representative staining panels of Ki-67 and MMP9 labeling in subcutaneous xenograft specimens in low-magnifying fields (100 ×). Then, percentage of Ki-67 (left) and MMP9 (right) staining was analyzed and positive cells with both markers in Folr1-Ara-C group were lower than others (*P < 0.01*). (**D**) MB-wyx cells were intracranially transplanted into nude mice, and microMRI scans on every week showed that Folr1-Ara-C markedly suppressed tumor growth. (**E**) PET/CT on 28th day showed that Folr1-Ara-C dramatically attenuated the metastasis. (**F**) Tumor volume curve showed that the xenograft in Folr1-Ara-C group progressed slowly. (**G**) SUV ratio (hypermetabolism center / natural background) and metastatic volume based on the PET/CT were shown and difference was significant (*P < 0.01*). (**H**) Kaplan-Meier curve for illustrating of the survival periods of the xenograft-bearing mice. (**I**) Representative images of Ki-67 and MMP9 expression in intracranial xenograft specimens in low-magnifying fields (100 ×). Percentage of Ki-67 (left) and MMP9 (right) staining was analyzed and there existed significant difference (*P < 0.0001*).

To extend our research achievements *in vitro*, we further investigated the functional relevance of Folr1-Ara-C in terms of MB growth and metastasis abrogating *in vivo*. Daoy and MB-wyx cells were subcutaneously injected into nude mice, respectively. Folr1-Ara-C durably impaired tumor growth as the tumor volumes remained the low levels throughout the treatment period (Figure 5A and 5B). By contrast, the tumor burden madly increased in control. Furthermore, immunoactivity to the proliferative marker, Ki-67 and aggressive marker, MMP9 was markedly diminished in tumors treated with Folr1-Ara-C compared with the Ara-C therapeutic group and control (Figure 5C). The morphology was shown in Supplementary Figure 5.

We consistently generated cohorts of MB-wyx cells bearing mice to assess the inhibiting effects on intracranial tumors. Representative microMRI scans for 3 distinct mice on every week showed that Folr1-Ara-C could enormously reduce the tumor burden, while faster tumor growth was found in the Ara-C administration group (Figure 5D and 5F). The most extensive metastasis was involved in the control and Folr1-Ara-C presented significant metastasis suppressing potential compared with the Ara-C according to the data of PET/CT on 28th day (Figure 5E). Then statistical graphs of standard uptake value (SUV) ratio and metastatic volumes (Figure 5G) displayed the more obvious potency of Folr1-Ara-C. In addition, mice treated with Ara-C exhibited a median survival of 31 days. Conversely the median survival of animals in Folr1-Ara-C group was prolonged to 42 days (Figure 5H). Moreover, we also generated the morphology of intracrinial xenografts (Supplementary Figure 5) and examined the percentages of Ki-67 and MMP9 labeling index showing that the ratios in the Folr1-Ara-C group decreased up to about 50% (Ki-67) and 60% (MMP9), respectively (Figure 5I). Taken together, these data suggested that Folr1-Ara-C might represent an effective target therapeutic application for MB.

## DISCUSSION

MB is the most common pediatric primary malignant brain tumor [1, 27]. Neither items have been regarded as the biomarkers for early diagnosis so for, nor items have been associated with neuroimaging characters. Data analysis of *folr1* gene amplification and mutation in cancers by cbioportal website (http://www.cbioportal.org) shows that Folr1 overexpressed in a wide range of malignant epithelial tumors. Recent research has illuminated the biological functions of Folr1 protein in many malignancies, but no studies are involved in MB. Herein we firstly confirmed the Folr1 expression in MB, consecutively identified its correlation with the clinicopathological and neuroimaging characteristics and finally developed the Folr1 targeted strategy for this devastating disorder.

In the current study, Folr1 was abundantly expressed in MB compared with the normal and benign tumors. Additionally, the MB samples with high Folr1 profiles showed the positive correlation with the percentage of Ki-67 and MMP9 labeling, suggesting that Folr1 played a valuable role in the malignancy prediction. Furthermore, Folr1 protein in large cell and anaplastic subtypes presented stronger expression than the other two subgroups, and the relevance of Folr1 with CSF spreading on MRI supported the evidence that patients with high levels tended to develop the neuro-metastasis.

Differential expression of Folr1 in pathological subgroups and risk grades is related to prognosis of MB patients, which can be recommended as a prognostic value. However, no significant difference exists in the OS of all the patients between Folr1-high and Folr1-low group in our study. The result is inconsistent with the report from Leung F [28]. The reasons may be that the follow-up period is not long enough to produce more accurate conclusion about OS and the data in TCGA is about mutation but not amplification. However, our study demonstrates that the pathological subtype can work as a prognostic index for PFS and OS in MB patients, which arrives at the same interpretation with Thompson EM [24].

The valuable tumor markers functioned as early diagnosis, therapeutic response monitoring, relapse prediction or prognosis evaluation for MB patients remain unknown in recent research. There have been a few studies regarding serum Folr1 as a biomarker for diagnosis in ovarian cancer [14, 29, 30]. Our data in ROC analysis suggests that serum levels of Folr1 exhibit more specific and accurate as an indicator for MB patients. Reasonably, Folr1 strictly locates in choroid plexus and data analysis of *folr1* gene in the Human Gene Database (http://www.genecards.org) also shows the high levels of Folr1 in normal CSF. Moreover, the Folr1 auto-antibodies or folate deficiency in CSF may lead to server development disorders of in children nervous system [31, 32]. Based on the above evidences, native Folr1 protein in CSF would produce interfering effects on the accuracy of examination in MB patients. Therefore, serum Folr1 can be utilized as a potential biomarker to evaluate the pathogenic condition, but not the CSF level. Given the limited quantity of samples, a great deal of data should be needed in the future study.

With the feature of unique expression in epithelial malignancies and restricted expression in normal tissues, Folr1 will be one of most promising target for treatment. In addition, FA as the legend for Folr1 shows a high-affinity of binding after derivatization and has been defined as the most widely investigated delivery for cancer therapy [33]. Moreover, only tumor-associated Folr1 can be accessible to FA in the circulation, but not the normal Folr1 [34]. A large number of imaging or treatment research have been involved in folate conjugates, including imaging agents [35, 36], chemotherapeutic agents [19, 37, 38], even siRNAs [39]. Our findings suggest that FA binding to Ara-C can be taken by Folr1-postive MB cells more effectively than Ara-C. Because the Folr1 targeted chemotherapeutic drug delivery can reduce the dosage of agents and side effects, increase the efficacy and specificity, as well as bypass the drug resistance, Folr1-Ara-C has shown promising application in a wide variety of cancers. By contrast, free FA would discount the anti-tumor capability by competitive binding to Folr1, which confirms the relationship between Folr1 and targeted treatment. Mechanistic investigations provide powerful consistence with the above results showing that Folr1 expression is positively correlated with proliferation and invasion. Folr1-Ara-C reduces the cell viability and mobility with down-regulation of MMPs proteins and activation of apoptosis-associated proteins. Previous study has also involved the molecular mechanisms of MMPs and Bcl-2/Bax proteins in repressing invasion and promoting apoptosis function of Folr1-linked agents [37, 40]. Beyond that, Folr1 inhibitors such as farletuzumab and IMGN853 are being investigated in some clinical trials [14, 41].

In conclusion, our study identifies the important role of Folr1 in MB and explains its correlation with clinicopathological characters and therapeutic target. The findings may provide the attractive diagnostic significance, including the value of tumor Folr1 as a molecular label in MB histopathology and the use of serum Folr1 as an indicator for pathogenic evaluation. Further we present the evidence supporting a novel application for target therapy in MB patients and even expand this strategy to other high-expressed malignancies.

## MATERIALS AND METHODS

### Patients and samples

The MB-associated specimens (tumor, serum and CSF) were obtained from 95 patients undergoing craniotomy. Pituitary adenoma, meningeoma (WHO-I), low-grade astrocytoma (WHO-II) and GBM (WHO-IV) specimens were obtained from 80 patients undergoing craniotomy, and normal cerebrum tissues were obtained from 6 patients undergoing cortex fistulation at Dept. of Neurosurgery in our institute from 09/2010 to 06/2015. All the diagnosis was confirmed by two experienced pathologists. None of tumors had been received chemo- and radiotherapy before operation. All tissues were collected during operation, then frozen and stored in liquid nitrogen. All procedures were approved by the Research Ethics Committee of China Capital Medical University (CCMU). Cell information can be found in the Supplementary Materials and Methods.

### Main agents

Folr1-Ara-C (5ml : 20mg, molecular weight 684.6) was purchased from CCMU. The compound was conjugated with FA, Ara-C and poly(ethylene glycol) (PEG) as previous described by Kim SH [42]. FA was reacted with PEG to synthesize the FA-PEG firstly and then the Ara-C was coated by the FA-PEG conjugates. The Folr1-Ara-C was further purified by a reversed-phas chromatography and freeze-dried at −20°C (Supplementary Figure 6). Ara-C (5ml : 100mg, molecular weight 243.2) was purchased from Pharmacia Italia of USA.

### Immunohistochemical staining

The IHC staining procedures were handled according to the manufacture's instructions. All the five-micrometer-thick sections were prepared from paraffin-embedded tissues. In brief, sections were stained with the anti-Folr1 antibody, anti-Ki-67 antibody and anti-MMP9 antibody (1 : 100) at 4°C overnight. Then the sections were washed and incubated using the secondary antibodies for 1 h. Normal brain tissues and placentas were used as negative and positive control, respectively. Evaluation was performed by analyzing the integrated optical density (IOD) on the images with the Image-Pro Plus 6.0 software or the percentage of positive staining. The expression of Folr1 was scored as follows: weak (IOD<10.00), moderate (10.00–15.00), strong (15.00–20.00) and very strong (> 20.00). Two experienced pathologists in our institute estimated the percentages of Ki-67^+^ cells or MMP9^+^ cells by counting the average number of stained cells per 500 nuclei in four high-magnifying fields (400×) randomly. Histological subgroups were also diagnosed by two pathologists in our institute.

### Flow cytometry analysis

The protocols for FCM analysis were based on the manufacture's recommendations and our previous work [43, 44]. In brief, Daoy and D283 cells were diluted 1 × 10^5^/ml, then treated with above agents for 8 h. The cells resuspended in 1 × binding buffer were incubated with Annexin V-FITC and PI in the dark for 20 min at room temperature, then analyzed by FACS. In addition, the prepared cells were resuspended in cold alcohol overnight at 4°C and then incubated in PI (50 ug/ml) with Triton X-100 in the dark for 30 min at 4°C. A minimum of 1 × 10^4^ cells with the gate region were analyzed by FACS to test the cell cycle.

### Western blot analysis

The whole protein (50 μg) was separated by 10% SDS-PAGE gel and then transferred to the PVDF membranes. The membranes were subsequently incubated with anti-Folr1, anti-EGFR, anti-pEGFR, anti-MMP2, anti-MMP9, anti-Caspase-3 anti-Caspase-9, anti-Bcl-2, anti-p53, anti-Erk, anti-pErk, anti-Stat3, anti-pStat3, and anti-β-actin antibodies (Sigma Biotechnology) overnight at 4°C. Secondary antibodies were used to probe the membranes for 1 h at room temperature. All of the first antibodies were diluted at 1 : 500 expect for β-actin at 1 : 3000.

### Animal and drug administration

All animal experiments were performed in accordance with national guidelines at CCMU and Chinese Academy of Medical Science (CAMS). Preliminary experiments showed that maximum tolerated dosage of Folr1-Ara-C and Ara-C was 10 mg/kg (subcutaneous injection) and 20 mg/kg (intraperitoneal injection). Daoy or MB-wyx cells (1 × 10^7^ / 100 μl) were subcutaneously injected into the right flank of six-to-eight-week-old male Bal-B/C nude mice. Seven days after transplantation, mice with comparable tumor burden were separated into four groups, Folr1-Ara-C, Ara-C, Folr1-Ara-C+free FA and control (*n* = 6 per group) and injected subcutaneously around the tumors every two days for another 3 weeks. Tumor volumes were measured every 3 days and calculated according to the following formula, V (mm^3^) = 1/2 (L × W^2^). After one month, the mice were sacrificed and tumor sections were stained with H.&E. and anti-Folr1, Ki-67 or MMP9 antibody.

MB-wyx cells (5 × 10^6^ / 5 μl) were intracranially injected into the right hemisphere, using a mouse stereotaxic apparatus at coordinates 1 mm posterior from the bregma, 1 mm lateral, and 3 mm ventral to the surface and delivered at a rate of 0.2 μl/min over 5 min. Engraftment was confirmed with microMRI scanning (on the 7th day after implantation) and mice with equivalent volumes were randomized into three groups: Folr1-Ara-C, Ara-C and control (*n* = 6 per group). Next, animals were treated by intraperitoneal injection every two days until they displayed tumor-associated morbidity. MRI scanning was performed once a week and PET/CT was also performed (on the 28th day) to investigate the metastasis. Tumor sections were stained with H.&E. and anti-Folr1, Ki-67 or MMP9 antibody.

### Statistical analysis

Data was shown as *mean ± SD* and statistically analyzed by one-way ANOVA, Student's *t*-test and Mann-Whitney test with SPSS 16.0 and Graphpad Prism 6.0. Correlations of Folr1 expression with clinicopathological characteristics were analyzed by Pearson or Spearman test. The risk factors were tested by Logistic regression analysis to estimate the odd ratio (OR). Survival data in the cohort was analyzed in the whole-case, average-risk and high-risk groups, respectively. Survival curves were performed by Kaplan-Meier method and the differences in OS or PFS were assessed through Log-rank test. Variate prognostic factors were analyzed by Cox proportional model. The *p* value of less than 0.05 was considered significance.

More detailed protocols were presented in the Supplementary Materials and Methods.

## SUPPLEMENTARY MATERIALS FIGURES AND TABLES



## Abbreviations

Folr1: Folate receptor 1
qRT-PCR: quantitative reverse transcriptase polymerase chain reaction
CT: computed tomography
MRI: magnetic resonance image
DMEM: dulbecco's modified eagle medium
FBS: fetal bovine serum
PTE: peritumoral edema
MMP2: Matrix Metalloproteinase-2
MMP9: Matrix Metalloproteinase-9
CSF: cerebrospinal fluid
TCGA: The Cancer Genome Atlas
H.&E.: hematoxylin & eosin
IC_50_: the half max inhibition concentration
MTS: methyl thiazolyl sulfide
FCM: flow cytometry
PI: propidium iodide.
