# Supplementary material for: Differential expression of folate receptor 1 in medulloblastoma and the correlation with clinicopathological characters and target therapeutic potential
Source: Oncotarget. 2017 Feb 18;8(14):23048–60. doi: 10.18632/oncotarget.15480 (PMC5410284; doi:10.18632/oncotarget.15480)
Supplement: Supplementary file 1 [file oncotarget-08-23048-s001.pdf]

Differential expression of folate receptor 1 in medulloblastoma and the correlation with clinicopathological characters and target therapeutic potential

Supplementary Materials

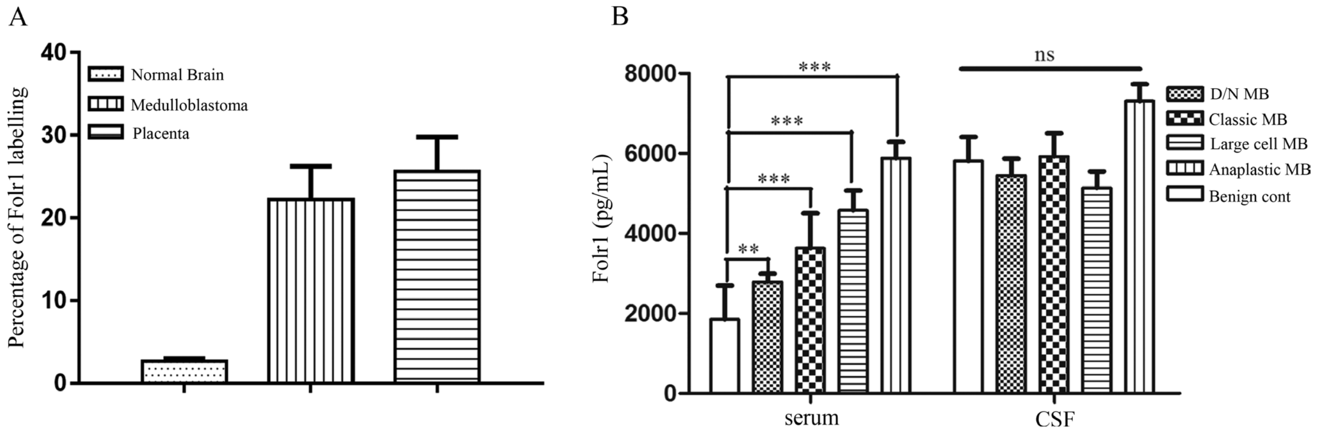

Supplementary Figure 1: (**A**) Statistic graph showed that Folr1 expression in MB was significantly higher than that in normal brain tissues ($P < 0.001$). (**B**) Serum and CSF Folr1 levels in benign meningioma control and different subgroups of MB. $^{**}P < 0.01$, $^{***}P < 0.001$.

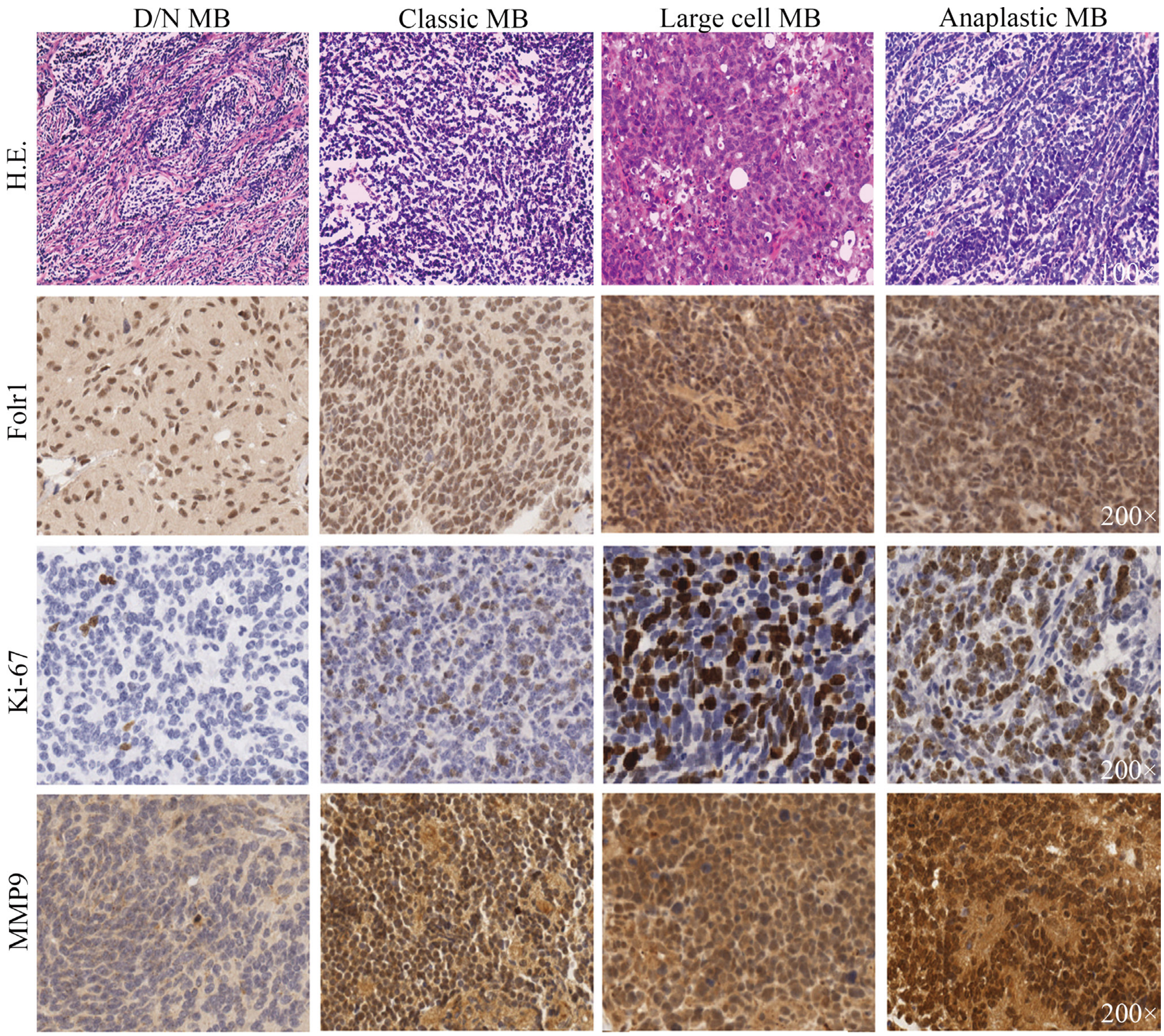

Supplementary Figure 2: Representative images of Folr1, Ki-67 and MMP9 labeling in human MB specimens in low-magnifying fields (100 ×). The first line showed the morphology of MB subtypes in high-magnifying fields (H.E., 200 ×).

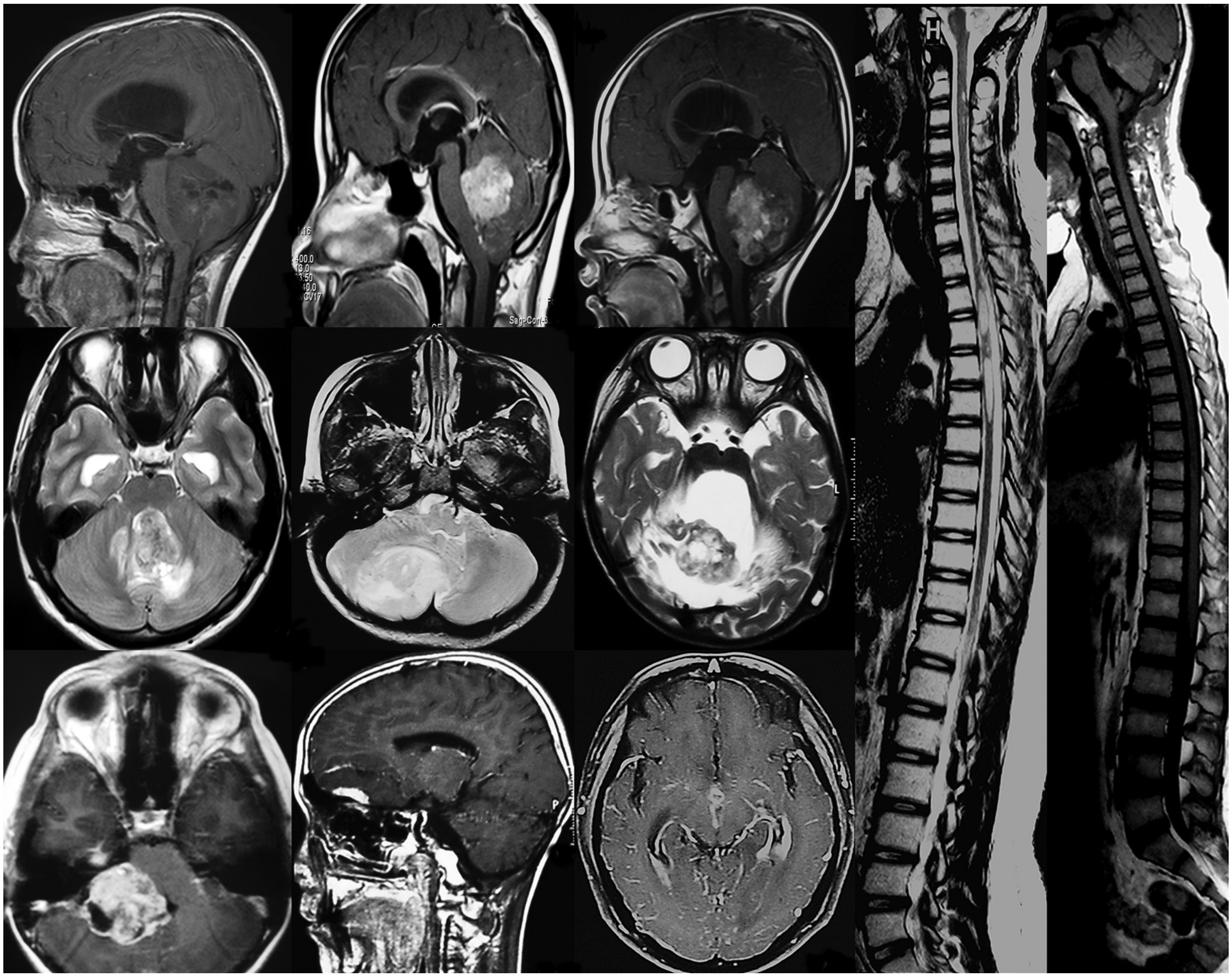

Supplementary Figure 3: Top, T1-weighted imaging with gadolinium showed enhancement was classified as non, heterogeneous or homogeneous features. Middle, T2-weighted imaging showed three grades of PTE. Down and right, cerebrospinal MRI showed tumor neuro-metastasis was classified as non- or CSF spreading.

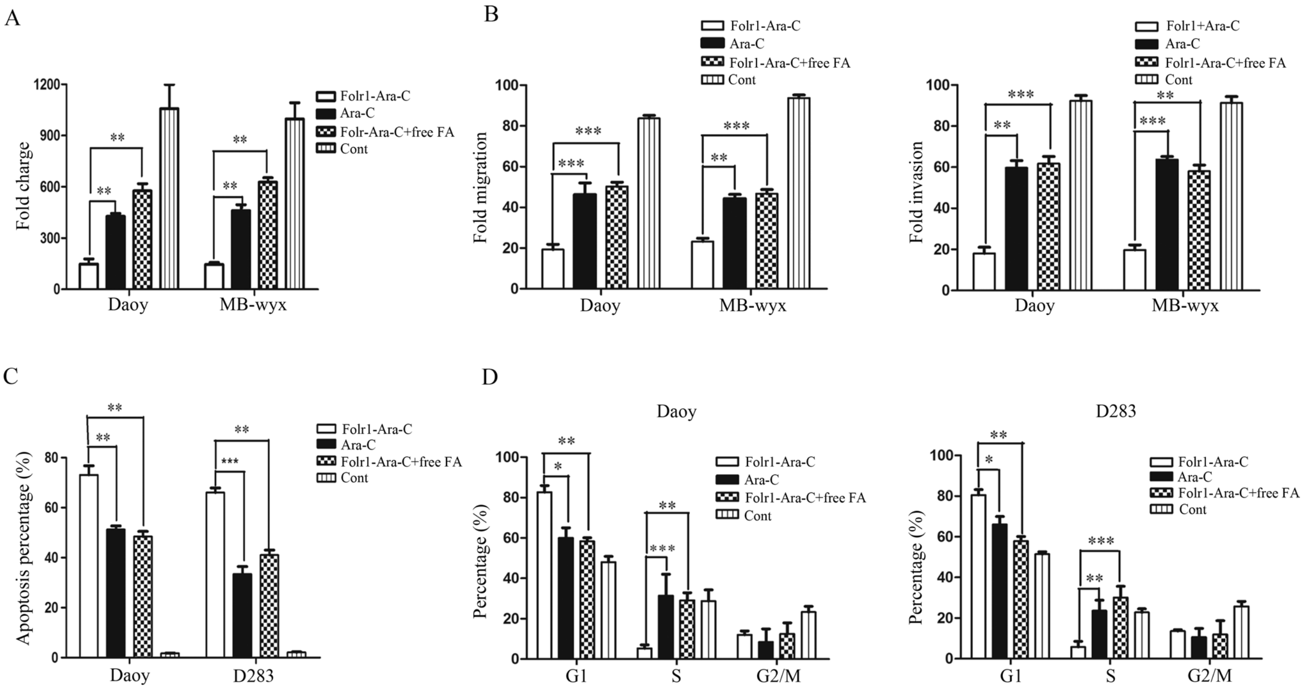

Supplementary Figure 4: (**A**) Statistic graphs of clonal formation assay. (**B**) Statistic graphs of cell migration and invasion. (**C**) Statistic graphs of cell apoptoasis. (**D**) Statistic graphs of cell cycle experiments. *$P < 0.05$, **$P < 0.01$, ***$P < 0.001$.

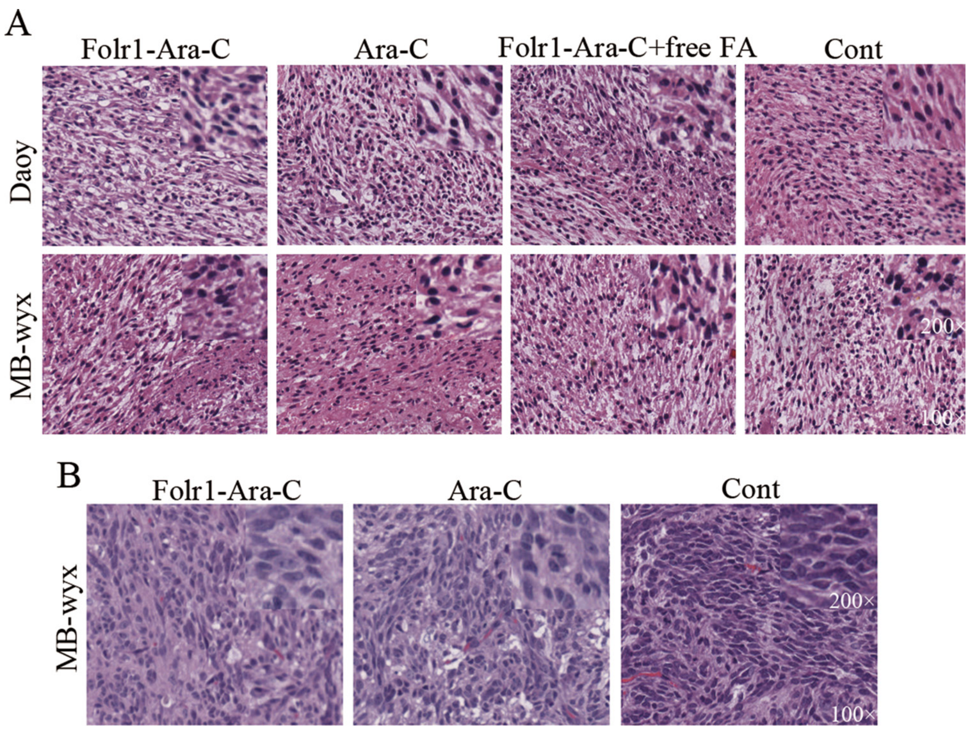

Supplementary Figure 5: (**A**) Representative H. E. staining images of Daoy (up) and MB-wyx (down) cell subcutaneous xenografts were shown in low-magnifying fields (100 ×) and high-magnifying fields (200 ×). (**B**) H. E. staining of MB-wyx cell intracranial xenografts were shown in low-magnifying fields (100 ×) and high-magnifying fields (200 ×).

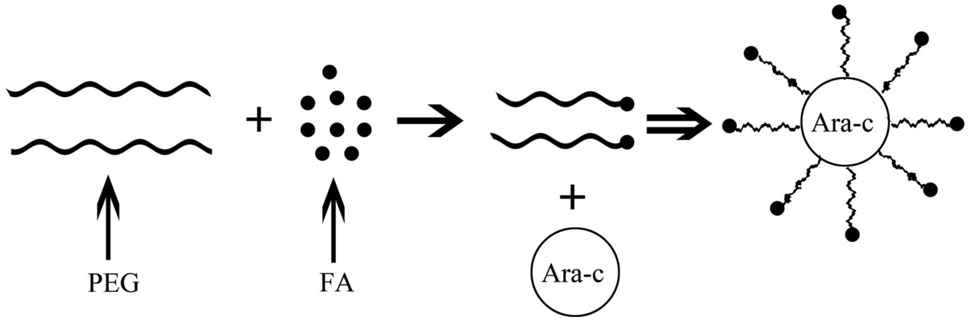

Supplementary Figure 6: The diagram of Folr1 targeted compound which showed that FA conjugated with Ara-C and PEG.

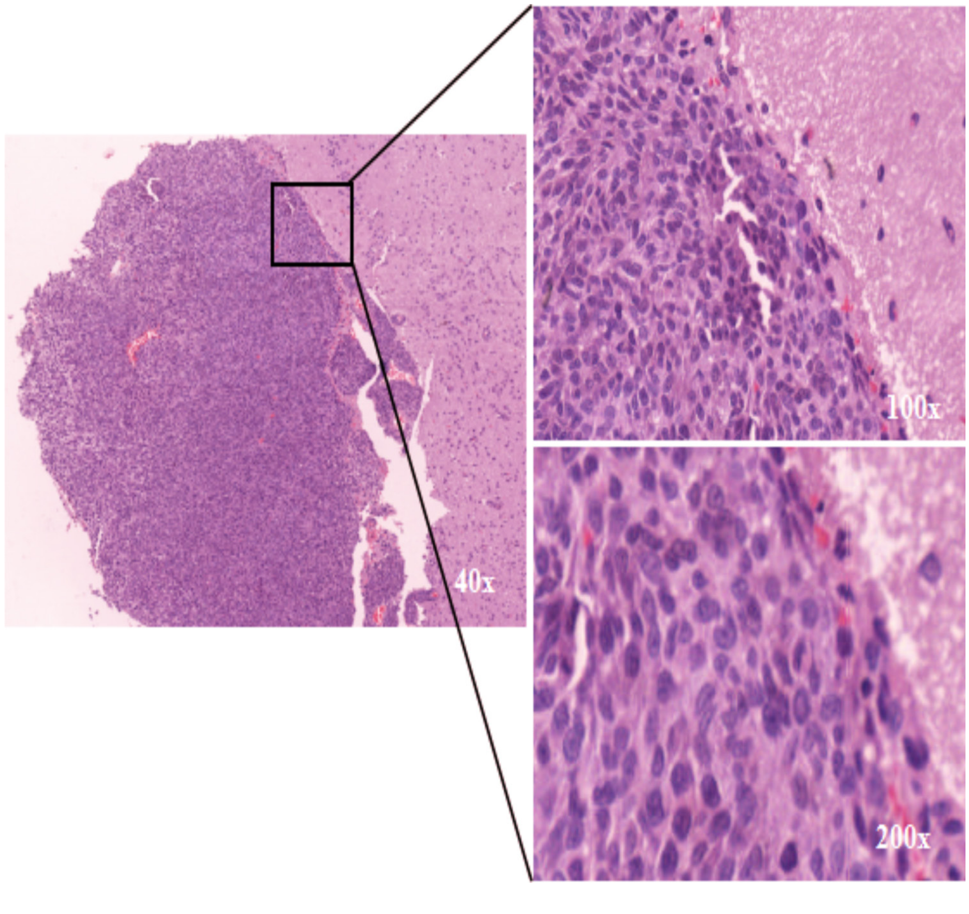

Supplementary Figure 7: Primary MB cells were isolated and the morphology of MB-wyx intracranial xenograft was shown in low-magnifying fields (40 ×, 100 ×) and high-magnifying fields (200 ×).

Supplementary Table 1: Correlation of Folr1 expression with clinicopathological and neuroimaging features in MB patients

Factors	No. (%)	Folr1 expression				*p* value	OR
		Weak	Moderate	Strong	Very strong		
		< 10.00	10.00-15.00	15.00-20.00	> 20.00		
**Gender**						0.04	
Male	56 (58.95)	22	9	11	14		
Female	39 (41.05)	10	19	8	12		
**Age (y)**						0.08	0.92
< 3	11(11.58)	1	1	2	7		
4–16	60 (63.16)	20	9	13	18		
> 17	24 (25.26)	11	6	4	3		
**Prognosis**						0.31	0.67
Alive	32 (33.68)	18	6	6	2		
Dead	63 (66.32)	16	10	13	24		
**Enhancement**						0.16	
No	3 (3.16)	2	0	0	1		
Heterogeneous	39 (41.05)	12	3	8	16		
Homogeneous	53 (55.79)	21	12	9	11		
**PTE**						0.14	
I	27 (28.42)	10	4	7	6		
II	43 (45.26)	8	12	9	14		
III	25 (26.32)	2	7	4	12		
**Dmax on MRI (cm)**						0.29	
< 2.9	18 (18.95)	8	2	4	4		
3–4.9	68 (71.58)	24	11	15	18		
> 5	9 (9.47)	0	2	4	3		
**CSF spreading**						< 0.001	2.73
Y	55 (57.89)	9	12	13	21		
N	40 (42.11)	25	4	6	5		
**Ki-67**						< 0.001	1.03
< 29.0%	22 (23.16)	21	0	1	0		
30.0–59.0%	42 (44.21)	11	17	11	3		
> 60.0%	31 (32.63)	0	0	7	24		
**MMP9**						< 0.001	1.15
Weak (< 19.0%)	12 (12.63)	12	0	0	0		
Moderate (20.0–29.0%)	46 (48.42)	20	14	9	3		
Strong (>30.0%)	37 (38.95)	1	2	11	23		
**Pathology**						< 0.001	1.10
D/N MB	4 (4.21)	3	1	0	0		
Classic MB	60 (63.16)	29	14	11	6		
Large cell MB	12 (12.63)	1	1	4	6		
Anaplastic MB	19 (20.00)	0	1	4	14		

Data was shown as number (percentage) of cases.

Supplementary Table 2: Summary ROC analysis for serum and CSF Folr1

	Serum Folr1	CSF Folr1
**AUC**	0.95 (0.92–0.99)	0.58 (0.44–0.74)
**Stan.Err**	0.02	0.08
***p* value**	< 0.0001	0.19
**Youden's index**	0.78	0.46
**Cut-off value**	2500.36 pg/mL	6850.05 pg/mL
**Sensitivity**	97.10%	45.70%
**Specificity**	88.10%	20.90%
**Accuracy**	88.57%	77.14%
**PPV**	82.93%	100.00%
**NPV**	96.55%	68.63%
**PLR**	8.68	0.58
**NLR**	0.03	2.60
***Kappa* value**	0.77	0.46

Ps: AUC = area under ROC curve, PPV = positive predictive value, NPV = negative predictive value, PLR = positive likelihood ratio, NLR = .negative likelihood ratio

Supplementary Table 3: Univariate and multivariate analysis by Cox proportional model

Factors	Univariate		Multivariate	
	HR (95% CI)	*p* value	HR (95% CI)	*p* value
**Sex (M/F)**	1.18 (0.59–2.38)	0.639		
**Age (≤ 3y/> 3y)**	2.20 (1.20–4.29)	0.004	1.19 (0.20–2.26)	0.003
**Enhancement (heter–/homo–)**	0.91 (0.44–1.86)	0.497		
**PTE (Low/High)**	1.86 (0.45–6.05)	0.603		
**Dmax on MRI (≤ 3 cm/> 3 cm)**	0.96 (0.33–2.80)	0.965		
**CSF spreading (Y/N)**	1.56 (0.26–1.76)	0.001	1.33 (0.14–1.76)	0.009
**Ki–67**				
< 29.0%	1 (Reference)			
30.0–59.0%	2.37 (0.82–6.91)	0.011	0.44 (0.06–3.85)	0.411
> 60.0%	1.93 (0.71–5.01)	0.002	1.46 (1.20–1.79)	< 0.001
**MMP9**				
Weak (< 19.0%)	1 (Reference)			
Moderate (20.0–29.0%)	2.49 (0.86–7.16)	0.911		
Strong (> 30.0%)	2.52 (1.03–4.08)	0.039	1.87 (0.63–3.58)	0.026
**Folr1 expression**				
Weak (5.00–10.00)	1 (Reference)			
Moderate (10.00–15.00)	1.60 (0.90–2.48)	0.712	1.47 (0.55–2.89)	
Strong (15.00–20.00)	2.20 (0.95–2.31)	0.018	1.96 (0.65–2.27)	< 0.001
Very strong (> 20.00)	2.28 (0.95–3.31)	0.06		
**Pathology**				
D/N MB	1 (Reference)			
Classic MB	2.01 (0.36–2.13)	0.006	0.91 (0.74–1.75)	0.118
Large cell MB	1.94 (0.35–3.73)	0.451		
Anaplastic MB	3.56 (1.96–4.29)	0.016	2.20 (0.88–3.16)	< 0.001

Supplementary Table 4: Log-rank test of OS dependent on the clinicopathological and neuroimaging variables of the cohort

Factors	No. (%)	Survival rate, No. (%)	Log-rank test
**Sex**			0.48
Male	56 (58.95)	17 (30.36)	
Female	39 (41.05)	14 (35.90)	
**Age**			< 0.001
< 3	11 (11.58)	4 (36.36)	
4–16	60 (63.16)	22 (36.67)	
> 17	24 (25.26)	6 (25.00)	
**Enhancement**			0.80
No	3 (3.16)	1 (33.33)	
heterogeneous	39 (41.05)	12 (30.77)	
homogeneous	53 (55.79)	19 (35.85)	
**PTE**			0.64
I	27 (28.42)	18 (66.67)	
II	43 (45.26)	10 (23.26)	
III	25 (26.32)	4 (16.00)	
**Dmax on MRI (cm)**			0.38
< 2.9	18 (18.95)	5 (27.78)	
3–4.9	68 (71.58)	23 (33.82)	
> 5	9 (9.47)	2 (22.22)	
**CSF spreading**			0.031
Y	55 (57.89)	12 (21.81)	
N	40 (42.11)	20 (50.00)	
**Ki–67**			0.009
< 29.0%	22 (23.16)	11 (50.00)	
30.0–59.0%	42 (44.21)	16 (38.90)	
> 60.0%	31 (32.63)	5 (16.13)	
**MMP9**			0.035
Weak (< 19.0%)	12 (12.63)	7 (58.33)	
Moderate (20.0–29.0%)	46 (48.42)	18 (39.13)	
Strong (> 30.0%)	37 (38.95)	11 (29.73)	
**Folr1 expression**			0.79
Weak (5.00–10.00)	33 (34.74)	17 (51.52)	
Moderate (10.00–15.00)	17 (17.89)	6 (35.29)	
Strong (15.00–20.00)	19 (20.00)	6 (31.58)	
Very strong (> 20.00)	26 (27.37)	3 (11.54)	
**Pathology**			0.042
D/NMB	4 (4.21)	4 (100.00)	
Classic MB	60 (63.16)	23 (38.33)	
Large cell MB	12 (12.63)	3 (25.00)	
Anaplastic MB	19 (20.00)	2 (10.53)	

Data was shown as number (percentage) of cases.

SUPPLEMENTARY MATERIALS AND METHODS

Cell culture

Human MB cell-lines, Daoy and D283, were purchased from Institute of Basic Medical Sciences Peking Union Medical College (PUMC). Primary GBM and MB cells, GBM-dzh and MB-wyx, and gliocytes were established from fresh specimens in our institute as described [2, 43] (Supplementary Figure 7). All the cells were maintained in DMEM supplemented with 10% FBS at 37°C in a humidified 5% CO_2 incubator and recently authenticated by cellular morphology. Cells in the following experiments were divided into four groups, Folr1-Ara-C, Ara-C, Folr1-Ara-C+free FA and 1 × PBS control.

Clinical and neuroimaging information

Clinical information was obtained from the records and follow-up materials. On CT and MRI scans, tumor size was graded as < 2.9 cm, 3.0–4.9 cm and > 5.0 cm according to the maximum diameter on axial contrast Gd-T1WI. Enhancement was classified as non, heterogeneous and homogeneous features according to the appearance on T1WI with gadolinium (Supplementary Figure 1B top). Peritumoral edema (PTE) on axial T2WI was analyzed on the basis of Asari's method [45–47] and defined as three grades: Gr I, diameter of PTE was less than that of tumor; Gr II, PTE diameter was more than or equal to tumor diameter but less than two times; Gr III, PTE diameter was two times more than or equal to tumor diameter (Supplementary Figure 1B middle). Tumor metastasis was classified as CSF spreading and non CSF spreading in accordance with cerebrospinal MRI [48] (Supplementary Figure 1B down and right). Two observers at the Neuroimaging Department of our institute independently interpreted the MRI scans.

Immunofluorescence staining

Daoy and MB-wyx cells were cultured on the dishes with bottoms coated by BD Matrigel Matrix at a density of 1×10^4/ well. After 48 h, the cells were incubated with blocking liquid for 1 h and subsequently incubated with anti-Folr1 antibody (1 : 100) overnight at 4°C and FITC secondary antibody at 25°C for 2 h.

RNA extraction and real-time RT-PCR

RNA was isolated from frozen cerebrum tissues (n = 6), pituitary adenomas (n = 18), WHO-I meningiomas (n = 12), Gr II. astrocytomas (n = 6), GBM (n = 15) and MB (n = 18) as well as Daoy, MB-wyx and GBM-dzh cells by using Trizol reagent according to the manufacture's procedures (Invitrogen, California). cDNA was synthesized by using iScript reverse transcription reagent from 2 μg of total RNA. The relative levels of mRNA were determined by using SYBR-green on the Mx3000P QPCR system and normalized for the expression of 18-S mRNA. Primer sequences were 5′-GTCGACACTGCTCATGCAAC-3′and 3′-AGGTTAAACGACAAGGACAGACA-5′. Real-time RT-PCR conditions were 95.0°C for 5 min, 35 cycles of 95.0°C for 30 s and 60.0°C for 30 s. Δσ Ct method was performed to calculate the fold induction and all the experiments were conducted three times [49].

ELISA

ELISA was performed using the MMP2, MMP9, cleaved Caspase-3/7 and -9 ELISA kits (Boster System) according to the manufacture's recommendations. Briefly, 100 μL of supernatant was added into duplicates and incubated at 37°C for 90 min. After washing 3 times, 100 μL of indicating antibodies was added and incubated at 37°C for 60 min. After color reaction with TMB substrate for 15 min, absorbency was detected at 450 nm in microplate reader (SpectraMax, US). The data was shown as mean ± SD at least three independent experiments.

Measurement of serum and CSF Folr1

Patients' serum and CSF samples were diluted at 1 : 10 concentration. The levels of Folr1 were measured by using the human Folr1 ELISA kit according to the above statements. According to the results, ROC curve was used to estimate the values with the SPSS 16.0. The values in the patients with benign meningiomas were used as the control. ROC curves illustrated the performance of trade-off existed between sensitivity and specificity for Folr1 protein in different groups. Youden's index was used in conjunction with ROC analysis to calculate the cut-off values in serum and CSF, which was defined for all the points in the ROC curves of serum or CSF. The maximum value of (sensitivity + specificity − 1) was considered as a criterion for selecting the optimal cut-off point.

Cell proliferation and colony formation assay

To identify the cell proliferation, the protocols and regents for MTS assay were strictly done according to our previous work [21]. 1×10^3 Daoy or MB-wyx cells were seeded in 60 mm dishes in DMEM with 10% FBS and cultured in the presence of experimental agents as indicated. After 10 days, the cells were immersed into 4% paraformaldehyde and 0.1% crystal violet.

Transwell assay

Migration and invasion assay were performed using the Transwell chambers. Briefly, 1×10^3 cells in 100 μL of serum-free DMEM were added into the upper chambers and cultured in the presence of experimental agents as above described, while the lower chambers were filled with 500 μL of DMEM with 10% FBS. After 8 h, the filters were fixed in methanol for 20 min and stained with 0.1% crystal violet, then counted. For the invasion assay, the upper chamber membrane was precoated with 50 μL 1.25 mg/ml Matrigel.
